# The Balloon-Based Manometry Evaluation of Swallowing in Patients with Amyotrophic Lateral Sclerosis

**DOI:** 10.3390/ijms18040707

**Published:** 2017-03-27

**Authors:** Jerzy Tomik, Barbara Tomik, Sebastian Gajec, Piotr Ceranowicz, Małgorzata Pihut, Rafał Olszanecki, Paweł Stręk, Jacek Składzień

**Affiliations:** 1 Ear, Nose & Throat (ENT) Department, Faculty of Medicine, Jagiellonian University Medical College, Kraków 31-531, Poland; j.tomik@uj.edu.pl (J.T.); sebastian.gajec@gmail.com (S.G.); pawel.strek@uj.edu.pl (P.S.); jacek.skladzien@uj.edu.pl (J.S.); 2 Department of Neurology, Faculty of Medicine, Jagiellonian University Medical College, Kraków 31-503, Poland; b.tomik@op.pl; 3 Department of Physiology, Faculty of Medicine, Jagiellonian University Medical College, Kraków 31-531, Poland; 4 Department of Prosthetic Dentistry, Faculty of Medicine, Jagiellonian University Medical College, Kraków 31-155, Poland; malgorzata.pihut@uj.edu.pl; 5 Department of Pharmacology, Faculty of Medicine, Jagiellonian University Medical College, Kraków 31-531, Poland; mfolszan@cyf-kr.edu.pl

**Keywords:** amyotrophic lateral sclerosis, dysphagia, bulbar onset, balloon-based manometry

## Abstract

The aim of the study was to analyse the disturbances of the oro-pharyngeal swallowing phase of dysphagia in amyotrophic lateral sclerosis (ALS) patients with the use of specific manometric measurements and to evaluate their plausible association with the duration of the disease. Seventeen patients with ALS were evaluated with manometric examinations of the oral and pharyngeal part of the gastrointestinal tract. Tests were carried out by using the oesophageal balloon-based method with four balloon transducers located 5 cm away from each other. The following manometric parameters were analysed: the base of tongue contraction (BTC) and the upper oesophageal sphincter pressure (UESP), and the hypopharyngeal suction pump (HSP) as well as the oro-pharyngeal, pharyngeal and hypopharyngeal transit time and average pharyngeal bolus velocity (oropharyngeal transit time (OTT), pharyngeal transit time (PTT), hypopharyngeal transit time (HTT) and average pharyngeal bolus velocity (APBV), respectively). Manomatric examinations during swallowing in patients with ALS showed significant weakness of BTC, a decrease of HSP and a decrease of the velocity of bolus transit inside the pharynx which were particularly marked between the first and the third examination. Manometric examinations of the oro-pharyngeal part of the gastrointestinal tract are useful and supportive methods in the analysis of swallowing disturbances in ALS patients.

## 1. Introduction

Abnormalities of deglutition in patients with amyotrophic lateral sclerosis (ALS) result from the weakness and discoordination of the muscles involved in the process of swallowing. Damage to the cranial nerves V, VII, IX, X and XII leads to abnormal mastication, inappropriate bolus formation and the impaired transit of the food bolus to the oesophagus. It has been shown so far that ALS patients suffer mainly from the abnormal oral phase of the swallowing (especially in case of predominant lower motor neuron damage) or the pharyngeal phase of the swallowing (particularly in case of predominant upper motor neuron damage) [1,2,3,4,5,6].

Damage to the lower motor neurons leads to the weakness of contraction within the base of the tongue, soft palate and muscles of the pharynx which results in the impaired tightness within the nasopharynx and regurgitation of food to the nasal cavity. Moreover, weakness of muscles that normally elevate the hyoid bone and larynx forwards and upwards decreases the opening of the upper oesophageal sphincter (UES), resulting in retention of the saliva, food and fluids within the piriform recess and the postcricoid area. Weakness of facial muscles (especially the orbicularis oris muscle) leads to the abnormal collection of saliva and precludes its removal from the oral cavity. Damage to the upper motor neurons results in the contraction of the cricopharyngeus muscle (being a part of the UES) or to its premature closure which consequently leads to the substantial impairment of the food passage, the retention of the food in pharynx and its aspiration to the respiratory tract.

Manometric parameters related to the passage of the bolus through the pharynx and its velocity are rarely reported in patients with ALS. Robbins [7] analysed disturbances of swallowing among 31 patients with ALS (including 22 with bulbar symptoms). He showed in these patients a prolonged time of transit of the food bolus during the oral phase of swallowing, during the time of disease, while patients without bulbar symptoms did not reveal such an abnormality. Similar findings were reported by Higo et al. [8] who used videofluoroscopy and showed decreased swallowing pressure, both in the oral cavity and in the hypopharynx, which correlated with the disease progression.

The aim of the study was to assess the disturbances of the oral and pharyngeal phase of swallowing in patients with bulbar form of ALS, focused on the determination of the type, dynamic and duration of the disease. An objective description of swallowing disturbances in ALS could be important for the evaluation of the severity insufficiency among these patients, i.e., for their prognosis.

## 2. Results

The patient group consisted originally of five (29%) men and 12 (71%) women (mean age at entry: 54.5 ± 11.4 years (range: 23–76)). The mean age at onset of symptoms was 53.3 ± 8.5 years (range: 31–74) and the average interval between the onset of symptoms and the first examination within this study was 31.4 ± 17.5 months. Forty-seven patients (73.4%) were diagnosed with limb-onset ALS and a further 17 (26.6%) subjects were diagnosed with the bulbar form of the disease.

Patients who could be examined only once because of subsequent death (*n* = 3:2 men and one women) or poor general health status (*n* = 3:1 men and two women) had a mean age of 62 ± 4.6 years and the duration of their disease at baseline ranged between 14 and 40 months (mean: 23.8). An additional four patients (one men and three women) who were examined twice and died before the third assessment were 64, 62, and 58 years of age, respectively, and at baseline, they had suffered from ALS for 28, 34 and 38 months, respectively.

### 2.1. Analysis of Results Obtained in Patients with ALS

Table 1 provides data on the measured manometric parameters during dry swallowing in patients with ALS and controls. Table 2 shows the same data for the wet swallowing.

In all three examinations, the mean values of the maximum BTC during dry and wet swallowing were decreased in patients with bulbar ALS. The mean value of resting UESP in patients with ALS was similar to that in the controls in the first and second examination only. In the third examination, a significant increase of UESP was noted among ALS patients in comparison with the controls during dry swallowing, but it was decreased in patients during wet swallowing. Both HSP and average pharyngeal bolus velocity (APBV) during dry and wet swallowing were substantially decreased among patients with ALS. Oropharyngeal transit time (OTT) and hypopharyngeal transit time (HTT) were markedly longer among patients than in controls, both during dry and wet swallowing.

### 2.2. Evaluation of Progression of Swallowing Disturbances among Patients with ALS

Mean values of particular parameters assessed during swallowing studies were compared in patients regarding the progression of abnormalities during the course of the disease. Table 3 shows data obtained during dry swallowing and Table 4 provides data recorded during wet swallowing.

### 2.3. Dry Swallowing

BTC decreased markedly between the first and third examination (*p* = 0.007) and between the second and third examination (*p* = 0.026). UESP values increased between the first and third examination, as well as between the second and third one (*p* = 0.005). HSP significantly decreased between the first and second examination, as well as between the second and third one (*p* = 0.05 and *p* = 0.006, respectively). APBV decreased significantly between the first and third (*p* = 0.006), second and third (*p* = 0.001) and between the first and second examination (*p* = 0.036).

OTT was significantly prolonged in the third examination when compared with the first one (*p* = 0.013), as well as between the first and second examination (*p* = 0.045). HTT was significantly longer in the third examination when compared with the first one (*p* = 0.002).

### 2.4. Wet Swallowing

BTC values were significantly lower in the second examination when compared with the first one (*p* = 0.004), in the third examination versus the first one (*p* = 0.001), as well as in the third examination when compared with the second one (*p* = 0.041). Resting UESP was markedly higher in the third examination when compared with the first (*p* = 0.004) or second one (*p* = 0.009). HSP was significantly decreased in the third examination when compared with the second one only (*p* = 0.05), while APBV was lower in the third examination when compared with the first one (*p* = 0.007) and lower in the second examination in comparison to the first one (*p* < 0.001). Mean OTT and HTT did not change significantly over the course of the disease.

## 3. Discussion

The importance of tongue movement in the mechanism of swallowing is highlighted by many authors [9,10,11]. Firstly, it takes part in the preparation and fragmentation of the food. Then, it is involved in the passage of the food bolus from the anterior part of the tongue towards its base. Finally, it moves the base of the tongue backwards, pushing it to the posterior wall of the pharynx, thereby increasing the BTC. This pressure plays a very important role in the further process of the swallowing act because it provides the driving force for the transit of the food bolus to the laryngeal part of the pharynx and to the oesophagus [12,13].

The available literature concerning the assessment of BTC in patients with ALS contains the report of Goeleven et al. [14], who performed manofluorographic examinations in 40 patients and evaluated the tongue driving force as well as the pharyngeal muscle contraction. They confirmed the decrease of the tongue driving force and the lowering of the amplitude of the pharyngeal contraction.

Kawai et al. [3] studied 11 patients and found two factors responsible for the swallowing abnormalities in the initial phase of dysphagia, namely the abnormal functioning of bolus transit from the anterior part of the tongue to its base and the detention of the bolus in the posterior part of the tongue.

Our study related to the assessment of BTC confirmed the presence of a marked decrease of BTC in patients with the bulbar onset of the disease. This abnormality was seen in both types of swallowing and deteriorated markedly between the first and third examination, i.e., after one year of follow-up.

UESP at rest and HSP altogether with the elevation of the larynx and its shift forwards are the most important factors responsible for the second phase of swallowing. Appropriately fast and efficient relaxation of the UES leads to its opening and results in the passage of the bolus to the oesophagus. Owing to the opening of UES, the pressure within its lumen decreases and produces HSP. The appropriateness of those parameters is closely associated with the motor function of the larynx. If the latter is compromised, the HSP could be decreased, and the UES opens incompletely or closes prematurely. All those factors lead to food retention in the piriform recesses and its possible aspiration to the larynx and respiratory tract [12,15,16].

Our study among patients with the bulbar form of the disease showed that resting UESP increases, especially after six and 12 months of follow-up, particularly in patients in whom wet swallowing was tested. HSP, however, was significantly lower in those patients. Based on the results presented here, we can suggest that HSP is very important in normal swallowing. Abnormal HSP values recorded in patients with ALS are seen earlier than changes in UESP and have a greater impact on the swallowing disturbances among those patients.

Cerenko et al. [17] believe that the presence of negative pressure within the UES substantially protects against the aspiration of food to the respiratory tract. The protection of the respiratory tract is provided not only by the mechanism closing the entrance to the larynx but also markedly by the negative pressure that sucks the food bolus to the oesophagus.

On the other hand, studies by Yokayama et al. [18] highlight the mechanism related to the laryngeal movement and its important role in swallowing. Those authors claim that the elevation of the larynx produces a shift of the epiglottis and closes the entrance to the larynx, while anterior movement of the larynx results in negative pressure within the UES and helps in the passage of the bolus to the oesophagus.

Studies related to the UES function in patients with ALS obtained divergent results. Kawai et al. [3] performed manometric studies in 11 ALS patients and found a decreased value of HSP in one patient only. Goeleven et al. [14] did not find any significant difference in that parameter between ALS patients and controls. The comparison of our results with those available in the literature is difficult because of the rarity of publications concerning this problem. It is also impossible to assess exactly the same parameters in functional examinations of the pharyngo-oesophageal segment. Nevertheless, Higo et al. [8], who used manofluorometric tests, strongly support the view that the pressure within the hypopharynx in ALS patients with dysphagia remains at a relatively similar level for a long time, and its decrease occurs about one year after the onset of symptoms. The same authors [19] performed a longitudinal analysis of disease progress among ALS patients and noted that the opening function of the UES remains unchanged for a long time during the course of the disease, thereby confirming our observations related to the earlier occurrence of HSP disturbances in relation to the pressure changes within the UES.

A manometric study performed by MacDougall et al. [4] in 13 patients with ALS did not confirm the presence of increased pressure within the UES or its abnormal relaxation, either in patients with dysphagia or in those with the features of food retention within the hypopharynx.

Wilson et al. [20] combined the UES with the motor function of the tongue. Among 27 patients diagnosed with the motor neuron disease, 13 had normal UES function. In seven patients with good or moderate motor function of the tongue (assessed visually and with the measurement of its elevation during pressing down), myotomy was subsequently performed. Those authors suggested that patients with weak motor function of the tongue poorly prepare the food bolus and move it to the pharynx with low force. Consequently, this abnormality does not release pharyngeal reflex and relaxation of the UES.

Videofluoromanometric studies performed by Higo [8] confirmed the presence of UES spasm in seven subjects (25.9%) among 27 patients with ALS. It is worthy to note that this symptom did not correlate with disease progression and with the increasing severity of other symptoms, but it was the reason for the aspiration of the food to respiratory tract in 57% of patients.

In the present study, we did not observe unequivocal UES spasm, but we did note the increase of the resting pressure only, which was much higher in patients with swallowing disturbances in the third examination in comparison with the first and second ones. It seems reasonable, therefore, that the UES dysfunction plays a minor role in the origin and mechanism of swallowing abnormalities among patients with ALS, while the pathology of the oral phase of swallowing seems to have a major role. Those findings are corroborated by Briani et al. [21], who use that notion to explain the divergent results achieved with UES myotomy in those patients, as reported in the literature [20,22,23,24,25].

Electromyographic studies of muscles involved in the swallowing process, published by Ertekin et al. [26], could serve as a summary of the problem of abnormal UES and hypopharynx function in the swallowing act. Those authors believe that the activity of muscles elevating the larynx during the swallowing act is decreased. It results probably from the weakness of the tongue and submental muscles because of the damage to the upper or lower motor neurons (or both). It leads to the transit of the bolus from the oral cavity to the respiratory tract before the swallowing reflex starts. It results also in the delayed opening and premature closure of the UES. It is due to the central inhibition rather than the action initiated by the peripheral nervous system.

Kristmundsdottir et al. [27] suggest that dysphagia in ALS results from the overlap of disordered central nervous system (CNS) action leading to the poor coordination of the pharyngo-oesophageal segment in patients with ALS.

Yokoyama et al. [18] studied that parameter and found that it depended largely on the BTC and the bolus consistency—a decrease of the BTC resulted in the elongation of the PTT. It corroborates with the studies performed by Mc Connel [13], who draws a comparison of the tongue action to the piston that drives the transit of the food bolus within the chamber delineated by the pharyngeal wall.

Thus, the majority of reports showing abnormal PTT concern patients after surgical treatment of malignancies within the larynx, oropharynx and laryngopharynx. It is supported by the results published by Walther [12], suggesting a marked elongation of the PTT value in those patients.

Our research confirmed the above-mentioned reports. The APBV was substantially lower, while the PTT was prolonged among patients with bulbar symptoms of the disease and in the oro-pharyngeal segment of the bolus passage.

## 4. Materials and Methods

### 4.1. Subjects

The study originally included 17 patients (five men and 12 women) with definite (three subjects, 17.6%) or probable (14 subjects, 82.4%) ALS diagnosed in the out-patient clinic for patients with ALS affiliated to the Department of Neurology, Jagiellonian University Medical College in Krakow. The diagnosis of definite or probable ALS was established by the same neurologist (BT) according to the El Escorial World Federation of Neurology criteria (1998).

Inclusion criteria consisted of the informed consent of subject to participate and the age between 18 and 80. Exclusion criteria comprised the family history of ALS, swallowing difficulties resulting from conditions other than ALS, anatomical conditions of lips, oral cavity or pharynx that precluded objective testing, as well as swallowing difficulties assessed in Hillel scale as five points, i.e., the use of liquefied diet [28]. Lack of informed consent to participate or clinical worsening precluding either attendance for follow-up examinations or the examination itself were also reasons for exclusion from the study.

Control group for the manometric studies of the upper part of the gastrointestinal tract consisted of 30 volunteers (20 men and 10 women) at the mean age of 53.7 ± 9.5 years (range: 39–71 years). Controls were free of any detectable laryngeal disorders; they had no disorders related to anatomical abnormalities of oral cavity, pharynx or larynx.

Study protocol was approved by the Bioethical Committee of Jagiellonian University (decision no. KBET/145/B/2006). All subjects provided their informed consent to participate in the study.

### 4.2. Methods

Manometric examination was performed three times in each patient, i.e., at the baseline, and approximately 6 and 12 months after the first evaluation. All three examinations were performed in seven patients (41.2%). Three patients died before the term of the second examination, and further three patients could not be examined owing to the poor general health status, immobilisation, or increased severity of symptoms precluding testing. Four other patients died before the third examination.

Manometric examination of the upper part of gastrointenstinal tract was performed with the use of Polygraf ID apparatus (Medtronic). Data were registered with the oesophageal tube Air Charge (Latitude^®^, London/Enfield, UK) equipped with four balloon pressure sensors positioned in 5 cm intervals. All data from each examination were entered to the computerised database with Polygram 98/Polygram Net software (Medtronic Gastroenterology, San Antonio, TX, USA).

The study was performed in the sitting position according to the technique described by Castell and Castell [29]. The oesophageal tube was positioned into the oesophagus transnasally. Local anaesthesia with 2% lignocaine was not administered to avoid the possible registration of distorted data owing to the abnormal food passage within the anesthetised oro- and hypopharynx. Pressure sensors (balloons) were positioned as follows: first—at the tongue base, second—at the entrance to the larynx (piriform recess), third—at the level of UES, and the fourth—in the cervical part of the oesophagus. Each subject performed five dry swallowing manoeuvres (saliva only) and five wet swallowing manoeuvres (about 10 mL of water per each swallowing act).

The following parameters were measured: (1) Base of tongue contraction (BTC), expressed in mm Hg, being the pressure value produced by the backward movement of the base of tongue and its contact with posterior wall of the pharynx during the oral phase of the swallowing; (2) UES pressure (UESP), expressed in mm Hg; (3) Hypopharyngeal suction pump (HSP), expressed in mm Hg × sec, being the negative pressure measured in the postcricoid segment as the area limited by the curve of the negative pressure in UES and the pressure level of 0 mm Hg; (4) Average pharyngeal bolus velocity (APBV), expressed in cm/s, reflecting the time of bolus transit through pharynx at the distance of 10 cm (i.e., between first and third balloon sensor); and (5) Pharyngeal transit time (PTT), expressed in seconds, being the time of bolus transit through the 10 cm segment between the oropharynx and the UES. The last parameter is divided into two parts; oropharyngeal transit time (OTT)—time needed for the bolus transit between first and second balloon sensor, i.e., from the base of tongue to the entrance to the larynx (5 cm long) and hypopharyngeal transit time (HTT) between second and third balloon sensor, i.e., between entrance to the larynx and UES (5 cm long as well). Both BTC and UESP were measured according to the description provided by Walther [12], while HSP, APBV, OTT and HTT were assessed in accordance with the protocol used by Mc Connel [9].

### 4.3. Statistical Analysis

Statistical analysis used non-parametric tests as the assumption of the normal distribution of variables was not met. Mann-Whitney test was used to compare quantitative variables (e.g., BTC) between patients and controls. Repeated measures within the studied group were analysed with Wilcoxon test. A *p*-value of ≤0.05 was considered statistically significant. Statistical analysis was performed with the use of STATISTICA v. 8.0 PL (StatSoft Polska, Kraków, Poland).

## 5. Conclusions

Manometric examination of the upper part of the gastrointestinal tract is an effective method for the detection of abnormalities during the oral and pharyngeal phase of swallowing in patients with ALS. This study confirms the presence of substantial and severe disturbances of the swallowing act, including the abnormal base of tongue contraction, the hypopharyngeal suction pump and the time and velocity of bolus transit through the oral cavity and pharynx.

Manometry of the upper part of the gastrointenstinal tract is a useful method in the assessment of the severity of deglutition disorders among patients with ALS.

## Figures and Tables

**Table 1:** Comparison of manometric parameters between patients with amyotrophic lateral sclerosis (ALS) (examinations I–III) and controls during dry swallowing (data are presented as means, standard deviations and ranges).

Variable	Patients with ALS			Controls (*n* = 30)	*p*-Value
	1st Examination (*n* = 17)	2nd Examination (*n* = 11)	3rd Examination (*n* = 7)		
Base of tongue contraction (mm Hg)	100.56 ± 27.07	98.95 ± 18.73	81.86 ± 12.58	161.04 ± 23.65	<0.001 ^a^
	(43.16–176.04)	(83.56–151.54)	(57.48–96.66)	(130.08–198.58)	
Upper oesophageal sphincter pressure (mm Hg)	55.48 ± 4.87	54.81 ± 6.05	61.76 ± 9.71	56.09 ± 4.33	<0.001 ^a^
	(43.78–64.58)	(56.18–64.86)	(45.64–71.78)	(51.28–68.82)	
Hypopharyngeal suction pump (mm Hg × s)	2.24 ± 1.22	1.69 ± 0.95	1.49 ± 0.90	3.39 ± 0.87	NS ^b^
	(0.49–4.28)	(0.42–3.48)	(0.39–3.20)	(2.34–5.92)	0.011 ^c^
Average pharyngeal bolus velocity (cm/s)	4.74 ± 1.17	3.96 ± 0.91	3.46 ± 1.13	7.57 ± 1.65	<0.001 ^a^
	(2.78–7.33)	(2.57–5.78)	(2.35–5.38)	(5.96–11.16)	
Oropharyngeal transit time (s)	0.95 ± 0.24	1.29 ± 0.44	1.41 ± 0.44	0.60 ± 0.13	<0.001 ^a^
	(0.61–1.64)	(0.95–2.44)	(0.88–1.98)	(0.36–0.75)	
Hypopharyngeal transit time (s)	1.35 ± 0.41	1.85 ± 1.17	1.85 ± 0.46	0.78 ± 0.16	<0.001 ^a^
	(0.76–2.06)	(0.91–5.15)	(1.16–2.36)	(0.54–1.04)	

^a^ For the difference between each examination in ALS patients and controls; ^b^ for the difference between first or second examination in ALS patients and controls; ^c^ for the difference between third examination in ALS patients and controls; NS—non-significant.

**Table 2:** Comparison of manometric parameters between patients with amyotrophic lateral sclerosis (ALS) (examinations I–III) and controls during wet swallowing (data are presented as means, standard deviations and ranges).

Variable	Patients with ALS			Controls (*n* = 30)	*p*-Value
	1st Examination (*n* = 17)	2nd Examination (*n* = 11)	3rd Examination (*n* = 7)		
Base of tongue contraction (mm Hg)	99.33 ± 18.54	91.37 ± 13.34	81.51 ± 13.76	134.25 ± 17.76	<0.001 ^a^
	(75.02–149.38)	(75.86–117.96)	(62.42–106.96)	(107.20–177.14)	
Upper oesophageal sphincter pressure (mm Hg)	52.65 ± 6.21	54.90 ± 5.70	52.65 ± 8.72	55.31 ± 4.45	0.048 ^b^
	(39.63–62.12)	(45.12–61.72)	(49.02–71.88)	(49.92–63.80)	NS ^c^
					0.001 ^d^
Hypopharyngeal suction pump (mm Hg × s)	1.27 ± 0.74	0.87 ± 0.50	0.76 ± 0.48	2.15 ± 0.57	<0.001 ^a^
	(0.40–3.22)	(0.23–1.72)	(0.28–1.72)	(1.40–3.30)	
Average pharyngeal bolus velocity (cm/s)	5.00 ± 0.84	4.03 ± 0.71	3.77 ± 1.01	7.46 ± 1.93	<0.001 ^a^
	(3.49–6.43)	(3.10–5.15)	(2.34–5.42)	(5.17–11.17)	
Oropharyngeal transit time (s)	0.97 ± 0.22	1.08 ± 0.17	1.04 ± 0.26	0.62 ± 0.14	<0.001 ^a^
	(0.71–1.59)	(0.87–1.43)	(0.74–1.55)	(0.32–0.77)	
Hypopharyngeal transit time (s)	1.41 ± 0.39	1.53 ± 0.27	1.66 ± 0.38	0.81 ± 0.18	<0.001 ^a^
	(0.81–2.12)	(1.11–1.87)	(1.20–2.41)	(0.50–1.22)	

^a^ For the difference between each examination in ALS patients and controls; ^b^ for the difference between first examination in ALS patients and controls; ^c^ for the difference between second examination in ALS patients and controls; ^d^ for the difference between third examination in ALS patients and controls; NS—non-significant.

**Table 3:** Progression of swallowing difficulties among patients with amyotrophic lateral sclerosis—dry swallowing.

Variable	Examinations Compared	*n*	Mean	SD	Absolute Difference	*p*-Value
BTC	I	11	101.55	11.59	−2.61	NS
	II		98.95	18.73		
	I	7	103.21	12.85	−21.35	0.007
	III		81.86	12.58		
	II	7	103.14	22.94	−21.28	0.026
	III		81.86	12.58		
UESP	I	11	56.12	5.73	−1.31	NS
	II		54.81	6.05		
	I	7	56.43	6.72	+5.33	0.005
	III		61.76	9.71		
	II	7	55.94	7.09	+5.82	0.005
	III		61.76	9.71		
HSP	I	11	2.28	1.14	−0.59	0.050
	II		1.69	0.95		
	I	7	2.39	1.12	−0.90	NS
	III		1.49	0.90		
	II	7	1.75	0.98	−0.26	0.006
	III		1.49	0.90		
APBV	I	11	4.21	0.80	−0.26	0.036
	II		3.96	0.91		
	I	7	4.23	0.93	−0.77	0.006
	III		3.46	1.13		
	II	7	3.92	1.10	−0.46	0.001
	III		3.46	1.13		
OTT	I	11	1.03	0.24	+0.26	0.045
	II		1.29	0.44		
	I	7	1.04	0.27	+0.37	0.013
	III		1.41	0.44		
	II	7	1.39	0.53	+0.02	NS
	III		1.41	0.44		
HTT	I	11	1.52	0.37	+0.33	NS
	II		1.85	1.17		
	I	7	1.53	0.41	+0.32	0.002
	III		1.85	0.46		
	II	7	2.08	1.43	−0.23	NS
	III		1.85	0.46		

SD—standard deviation; BTC—base of tongue contraction; UESP—upper oesophageal sphincter pressure; HSP—hypopharyngeal suction pump; APBV—average pharyngeal bolus velocity; OTT—oropharyngeal transit time; HTT—hypopharyngeal transit time; NS—non-significant.

**Table 4:** Progression of swallowing difficulties among patients with amyotrophic lateral sclerosis—wet swallowing.

Variable	Examinations Compared	*n*	Mean	SD	Absolute Difference	*p*-Value
BTC	I	11	100.98	12.84	−9.61	0.004
	II		91.37	13.34		
	I	7	104.77	13.41	−23.25	0.001
	III		81.51	13.76		
	II	7	91.89	14.84	−10.38	0.041
	III		81.51	13.76		
UESP	I	11	53.63	5.80	+1.27	NS
	II		54.90	5.70		
	I	7	54.23	6.09	+8.42	0.004
	III		62.65	8.72		
	II	7	55.95	4.97	+6.69	0.009
	III		62.65	8.72		
HSP	I	11	1.20	0.83	−0.33	NS
	II		0.87	0.50		
	I	7	1.49	0.89	−0.73	0.050
	III		0.76	0.48		
	II	7	0.95	0.49	−0.18	NS
	III		0.76	0.48		
APBV	I	11	4.91	0.89	−0.89	<0.001
	II		4.03	0.71		
	I	7	4.94	1.02	−1.17	0.007
	III		3.77	1.01		
	II	7	4.01	0.77	−0.24	NS
	III		3.77	1.01		
OTT	I	11	1.02	0.21	+0.05	NS
	II		1.08	0.17		
	I	7	1.05	0.26	−0.01	NS
	III		1.04	0.26		
	II	7	1.11	0.19	−0.06	NS
	III		1.04	0.26		
HTT	I	11	1.54	0.39	−0.01	NS
	II		1.53	0.27		
	I	7	1.55	0.41	+0.11	NS
	III		1.66	0.38		
	II	7	1.52	0.31	+0.14	NS
	III		1.66	0.38		

SD—standard deviation; BTC—base of tongue contraction; UESP—upper oesophageal sphincter pressure; HSP—hypopharyngeal suction pump; APBV—average pharyngeal bolus velocity; OTT—oropharyngeal transit time; HTT—hypopharyngeal transit time; NS—non-significant.
